# Practical Considerations for Odevixibat Treatment in Patients with Progressive Familial Intrahepatic Cholestasis: A Single-Center Case Series

**DOI:** 10.3390/jcm13247508

**Published:** 2024-12-10

**Authors:** Milena Marx, Steffen Hartleif, Johannes Hilberath, Christoph P. Berg, Ilias Tsiflikas, Stephan Singer, Ekkehard Sturm

**Affiliations:** 1 Pediatric Gastroenterology and Hepatology, University Children’s Hospital Tübingen, Hoppe-Seyler Str. 1, 72076 Tübingen, Germany; 2 Internal Medicine I, University Hospital of Tübingen, Otfried-Müller-Str. 10, 72076 Tübingen, Germany; 3 Department of Diagnostic and Interventional Radiology, University Hospital of Tübingen, Hoppe-Seyler Str. 3, 72076 Tübingen, Germany; 4 Institute of Pathology, Department for General and Molecular Pathology, University Hospital of Tübingen, Liebermeisterstrasse 8, 72076 Tübingen, Germany

**Keywords:** progressive familial intrahepatic cholestasis, odevixibat, serum bile acids, pruritus

## Abstract

**Background**: Patients with progressive familial intrahepatic cholestasis (PFIC) experience cholestasis-associated symptoms, including severe pruritus. Odevixibat is an ileal bile acid transporter inhibitor indicated for treatment of PFIC in the European Union and for the treatment of pruritus in PFIC in the United States. The aim of the current study was to characterize the real-world effectiveness and safety of odevixibat in patients with PFIC. **Methods**: This retrospective study included 9 patients with PFIC treated with odevixibat in a single center in Tübingen, Germany. Data were recorded using case report forms. **Results**: Of the 9 patients (PFIC1, *n* = 2; PFIC2, *n* = 7), 5 had improved serum bile acid levels, pruritus, liver function tests, and sleep with odevixibat treatment. Two siblings with periodic relapses of PFIC symptoms also had improved pruritus and sleep within 4 months of treatment. Two siblings with complete loss of bile salt export pump (BSEP) protein did not respond to treatment; both underwent liver transplantation (indications: hepatocellular carcinoma [HCC] manifestation [*n* = 1] and severe failure to thrive and refractory pruritus [*n* = 1]). Four patients reported abdominal complaints that were transient or responded to dose reduction; no other safety issues were reported. **Conclusions**: In this case series, clinical benefits were observed in most patients with PFIC1 and PFIC2 treated with odevixibat. In patients with periodic relapse of PFIC symptoms, ≥3 months of treatment with odevixibat may be required for symptom control. Patients with complete loss of BSEP did not have consistent symptom relief and require careful monitoring. Effectiveness and feasibility results from our cohort demonstrate potential for long-term benefits with odevixibat in real-world treatment of patients with PFIC.

## 1. Introduction

Progressive familial intrahepatic cholestasis (PFIC) is a group of rare cholestatic liver diseases caused by autosomal mutations in several genes, including *ATP8B1* (encoding familial intrahepatic cholestasis 1 [FIC1]; PFIC type 1 [PFIC1]) and *ABCB11* (encoding bile salt export pump [BSEP]; PFIC type 2 [PFIC2]) [1,2,3]. The nomenclature for these diseases is not uniformly defined. For the specific diseases discussed in our study, the following terms have been used synonymously: PFIC1 or FIC1 deficiency and PFIC2 or BSEP deficiency. For the purpose of clarity to the reader, we will use the terms PFIC1 and PFIC2 but are aware that the synonymous terms FIC1 deficiency and BSEP deficiency will find broader acceptance in future publications.

Patients with PFIC2 can be further classified by genotypically defined BSEP variant subtypes that are associated with phenotypes of variable disease severity: BSEP1 (least severe), BSEP2, and BSEP3 (most severe) [4]. PFIC1 and PFIC2 commonly present in early infancy; severity and progression of disease is often dependent on the degree of protein deficiency [1,2,4].

Cholestatic liver injury in PFIC is caused by impaired bile acid transport and flow leading to accumulation of bile acids in the hepatobiliary system, damage to hepatocytes and cholangiocytes, and inflammation and fibrosis [1,5]. These events can lead to end-stage liver disease and eventual need for liver transplantation [1,5]. Recent evidence suggests that less than 50% of patients with PFIC1 or PFIC2 reach adulthood with their native liver [4,6]. Additionally, build-up of bile acids in the systemic circulation may contribute to severe pruritus, which can impair sleep and quality of life for patients and their families [3,7,8]. Other PFIC characteristics include poor growth and fat-soluble vitamin deficiencies [1,2]. Patients with PFIC2, and particularly those with BSEP3 subtype mutations, have an increased risk for hepatocellular carcinoma (HCC) [2,4].

Historically, treatments for PFIC have been palliative and were aimed at nutritional support and pruritus relief [9]. Options for treating cholestatic pruritus include off-label therapies that have limited effectiveness in some patients (e.g., ursodeoxycholic acid [UDCA], rifampicin, cholestyramine) [9,10]. Alternative interventions to relieve intractable pruritus and liver disease include invasive procedures such as nasobiliary drainage (NBD) and surgical biliary diversion (SBD), both of which aim to reduce hepatic accumulation of bile acids, and liver transplantation [9,11]. The lack of effective, noninvasive treatments for pruritus in patients with PFIC has presented a clear medical need for novel therapeutics.

Odevixibat is an inhibitor of the ileal bile acid transporter (IBAT) that blocks the reabsorption of bile acids in the distal ileum and increases their excretion in the colon [12]. Odevixibat is indicated for the treatment of PFIC in the European Union and for pruritus in PFIC in the United States; in addition, odevixibat is indicated for the treatment of cholestatic pruritus in Alagille syndrome in the European Union and the United States [13,14,15]. Results from the phase 3 PEDFIC 1 and PEDFIC 2 studies of odevixibat in patients with PFIC have demonstrated that treatment with odevixibat improved pruritus and reduced serum bile acid levels (co-primary endpoints); improvements in liver function tests, sleep parameters, and growth were also observed [16,17]. Data on real-world efficacy and safety of odevixibat in patients with PFIC are currently limited.

Here, we describe real-world effectiveness, safety, and practical aspects of administration of odevixibat in everyday life in patients with PFIC treated at a single center in Tübingen, Germany. A plain-language summary of our findings has been included in the Supplementary Materials.

## 2. Methods

### Patients

This retrospective case series included 9 patients diagnosed with PFIC who were observed at University Children’s Hospital Tübingen, in Tübingen, Germany, over an observation period spanning from April 2008 to July 2023 and treated with odevixibat at the center. The earliest initiation of real-world odevixibat treatment in this patient cohort was in 2021. Some patients were additionally treated with odevixibat during participation in the phase 3 PEDFIC 1 clinical trial (ClinicalTrials.gov identifier: NCT03566238; submitted 25 May 2018; study record posted 25 June 2018) and/or the PEDFIC 2 clinical trial (ClinicalTrials.gov identifier: NCT03659916; submitted 24 August 2018; study record posted 6 September 2018); however, patient data during the treatment periods of these trials are not presented in this manuscript [16,17].

All patients in our cohort were diagnosed in childhood with compatible phenotypes of PFIC1 or PFIC2, and all diagnoses were confirmed by genotyping. Individual patient data were recorded by the treating physicians using a standardized case report form; in this form, pruritus severity during treatment with odevixibat was reported according to the assessment methods used by treating physicians, which could include use of a visual analog scale (VAS) (Figure S1).

Parental or guardian approval was obtained for anonymized publication of individual data.

## 3. Results

### 3.1. Patient Characteristics and Medical History

Table 1 shows the demographics, baseline characteristics, and medical history for the nine patients included in this case series. Of these nine patients, seven (78%) were male, two (22%) were diagnosed with PFIC1 and seven (78%) were diagnosed with PFIC2. Immunohistochemistry results from liver biopsies revealed no BSEP protein expression in three patients with PFIC2 (patients 1, 2, and 8). Patients 1 and 2 are siblings and were classified as having BSEP3 subtype mutations, while patient 8 was classified as having BSEP2 subtype mutations [4]. Although patients 8 and 9 are siblings with the same mutations in the *ABCB11* gene, a liver biopsy was not performed for patient 9, and BSEP protein expression was not determined. The median age at diagnosis was 8 months (range, 1 month to 15.1 years), and the median age at last assessment was 6.4 years (range, 1.3–22.9). All nine patients presented with low or normal gamma-glutamyl transferase (GGT) activity and variable elevations in aminotransferase and bilirubin levels.

All nine patients reported pruritus as a symptom (Table 1). Four patients had a history of neonatal cholestasis, seven patients had a history of jaundice, and two patients were diagnosed with vitamin K deficiency hemorrhage. Patient 9 was diagnosed with a coagulation disorder as a neonate and received vitamin K as needed. Patients 8 and 9 experienced periodic relapses of symptoms, and patient 9 had temporary placement of a nasobiliary tube to relieve pruritus during relapse. Patient 6 also had temporary placement of a nasobiliary tube to relieve pruritus symptoms. Patients 1 and 2 were both using percutaneous endoscopic gastrostomy (PEG) due to failure to thrive.

### 3.2. Odevixibat Treatment

The median observation time before start of any odevixibat treatment was 1.8 years (range, 0.3–14.4 years) (Table 2), and intractable pruritus was reported as the primary reason for starting odevixibat in all nine patients. Physicians reported that in 6 patients (patients 2, 3, 4, 6, 7, and 9), pruritus-associated sleep disturbance was also a reason for starting treatment. Failure to thrive was additionally reported as a reason for starting odevixibat for patients 1 and 2.

The median age at start of real-world odevixibat treatment was 5.5 years (range, 0.5–21.7 years) (Table 2). Five patients participated in PEDFIC 1 and/or PEDFIC 2 and received placebo and/or odevixibat during those studies. Median treatment duration outside of the PEDFIC trials [16,17] was 11 months (range, 5–15 months). Patient 9 stopped odevixibat treatment for 4 months due to cessation of symptoms and then restarted due to relapse.

**Figure 1:**
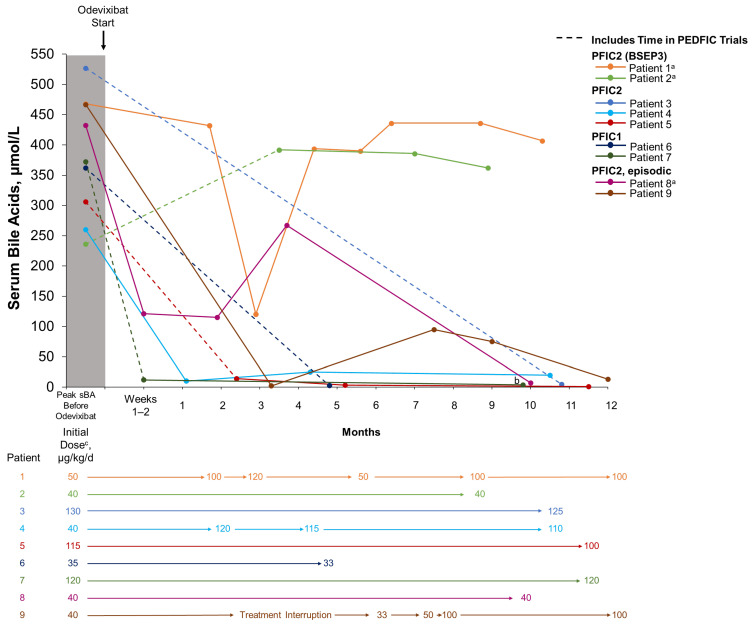
Serum bile acid levels and dosing over time with real-world odevixibat treatment. For patients who participated in the PEDFIC clinical trials, dotted lines indicate time spent in the PEDFIC trials as well as time during real-world odevixibat treatment. Solid lines indicate real-world odevixibat treatment. ^a^ No BSEP protein expression. ^b^ Nonfasting sBA was measured as 312 μmol/L at 14.6 months of treatment for patient 7. ^c^ Outside of PEDFIC trials. BSEP, bile salt export pump; PFIC, progressive familial intrahepatic cholestasis; sBA, serum bile acid.

Throughout the real-world treatment period, odevixibat doses ranged from 33 to 130 µg/kg/day (Table 2). Odevixibat dose was unchanged over time in six patients and was escalated in three patients during real-world treatment (patients 1, 4, and 9) (Figure 1). For three patients, the per-kg dose decreased due to weight gain (–4%, –6%, and –13% for patients 3, 6, and 5, respectively) (Figure 1).

The majority of patients (8/9) received concomitant anti-pruritic medications during treatment with odevixibat, including UDCA (patients 1, 2, 3, 4, 5, 6, 8, and 9) and/or rifampicin (patients 2, 4, 8, and 9). Patients 4 and 9 discontinued rifampicin use during treatment with odevixibat; patient 9 subsequently initiated use of UDCA.

### 3.3. Effectiveness and Safety with Odevixibat

**Figure 2:**
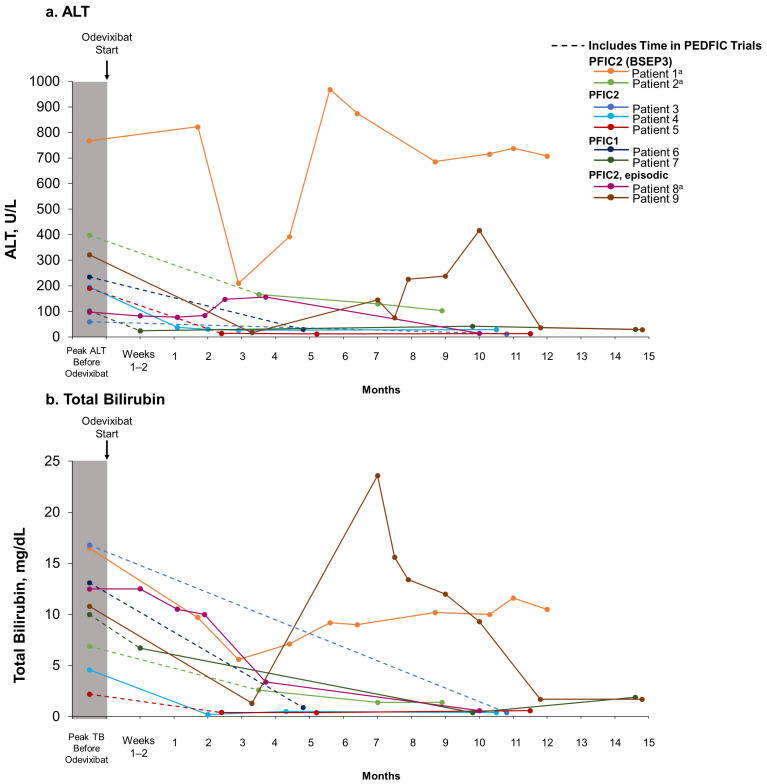
ALT (**a**) and total bilirubin (**b**) levels over time with real-world odevixibat treatment. For patients who participated in the PEDFIC clinical trials, dotted lines indicate time spent in the PEDFIC trials as well as time during real-world odevixibat treatment. Solid lines indicate real-world odevixibat treatment. ^a^ No BSEP protein expression. ALT, alanine aminotransferase; BSEP, bile salt export pump; PFIC, progressive familial intrahepatic cholestasis; TB, total bilirubin.

Patients 3–9 experienced reductions in serum bile acid levels (Figure 1) and improvements in pruritus and sleep disturbance (Table 3) with odevixibat treatment. Eight patients also experienced reductions in alanine aminotransferase (ALT) and total bilirubin with odevixibat (patients 2–9) (Figure 2). Patient 1, who had PFIC2 with BSEP3 subtype mutations, experienced consistent pruritus improvement without consistent reductions in serum bile acid levels or liver function tests while on odevixibat (Table 3, Figure 1 and Figure 2). Patient 2, also with BSEP3 subtype mutations, had low global satisfaction and no consistent pruritus improvement with odevixibat (Table 3).

All patients who responded to odevixibat and who had available weight and height Z scores (i.e., patients who initiated real-world odevixibat at age < 18 years) had scores within the normal range before starting real-world odevixibat (patients 3–5, 7, and 8; Table S1). Median changes in weight and height Z scores were −0.06 and −0.25, respectively, in these five patients at last assessment on treatment. Patients 1 and 2 with BSEP3 genotypes experienced failure to thrive in the course of their disease, with further decreases in Z scores for weight and/or height while on odevixibat treatment.

**Figure 3:**
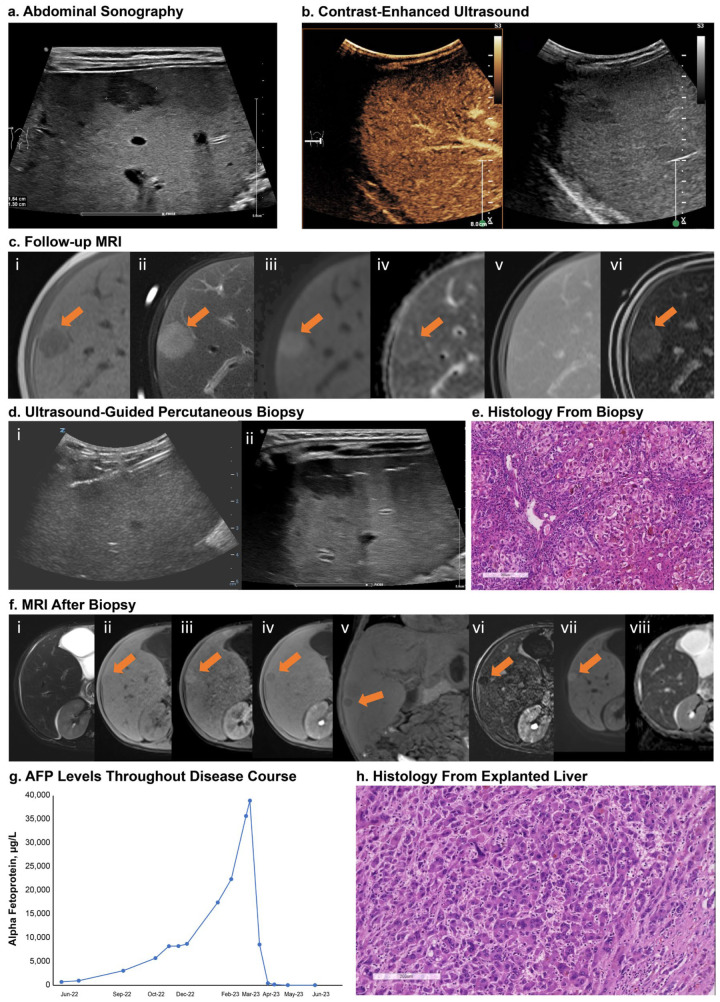
Screening for HCC in a patient with BSEP3 (patient 1). Panel (**a**): sonography showed focal liver lesions; largest legion in segment 7 with maximum diameter 1.5 cm. Panel (**b**): contrast-enhanced ultrasound showed enhancement of lesion without evidence of wash-out. Panel (**c**): liver MRI with gadobutrol showed hypointense signal on T1w (**i**) and hyperintense signal on T2w (**ii**). DWI showed increased signal at high *b*-values (**iii**) and slightly low signal intensity on ADC map (**iv**). Lesion showed enhancement on dynamic contrast-enhanced imaging without wash-out in the venous phase (**v**). Repeat MRI with a hepatospecific paramagnetic gadolinium-based contrast agent, gadoxetate disodium, also showed enhancement in the dynamic contrast-enhanced imaging without wash-out (**vi**). Panel (**c**(**vi**)): subtraction image of the late hepatobiliary phase taken at 25 min. Panel (**d**): visualization of the specific echogenic ultrasound needle and position of the needle tip in the lesion (**i**) and gas artifacts along the puncture canal (**ii**). Panel (**e**): H&E staining showed severe cholestatic hepatitis with giant cell transformation, hepatocellular and canalicular bilirubinostasis, ductular reaction, and inflammation. Panel (**f**): five-month follow-up MRI after biopsy showed a new lesion of 10 mm contiguous to the scar zone of the biopsy; slightly hyperintense on T2w (**i**) and hypointense on T1w (**ii**) with hyperarterialization on dynamic contrast-enhanced imaging (**iii**) and wash-out in the hepatobiliary phase ((**iv**)—transverse plane; (**v**)—coronal plane). The subtracted transverse plane of the hepatobiliary phase enhanced the wash-out phenomenon (**vi**). DWI showed increased signal at high *b*-values (**vii**) and low signal intensity on ADC map (**viii**). Panel (**g**): AFP levels. Panel (**h**): H&E staining of explanted liver showed hepatoid tumor cell infiltrates compatible with diagnosis of HCC. Scale bar = 200 µm. Arrows indicate lesion location. ADC, apparent diffusion coefficient; AFP, alpha fetoprotein; BSEP, bile salt export pump; DWI, diffusion-weighted imaging; H&E, hematoxylin and eosin; HCC, hepatocellular carcinoma; MRI, magnetic resonance imaging; T1w, T1 weighted; T2w, T2 weighted.

Patients 1 and 2 (siblings) underwent liver transplantation: patient 1 underwent liver transplantation due to HCC manifestation, and patient 2 due to severe failure to thrive and refractory severe pruritus. Patient 1 was screened for suspected HCC due to focal liver lesions detected in an abdominal sonography conducted as part of standard follow-up (Figure 3a). Contrast-enhanced ultrasound (Figure 3b) and magnetic resonance imaging (MRI) (Figure 3c) of the liver were performed and showed enhancement of the largest lesion (segment 7, maximum diameter: 1.5 cm). An ultrasound-guided percutaneous biopsy was performed (Figure 3d), and histopathology showed cholestatic hepatitis with giant cell formation and single cell and group necrosis and fibrosis compatible with a diagnosis of advanced cholestatic infantile liver disease. No evidence of malignancy was found. A second, follow-up MRI using a hepatospecific contrast agent confirmed the initial findings (i.e., enhancement of the lesion without wash-out; Figure 3c(vi)). Due to the MRI results and the patient’s increasing alpha fetoprotein (AFP) levels (Figure 3g), an open surgical biopsy was recommended for histological confirmation of the findings. Histopathology again showed cholestatic liver parenchyma with advanced fibrosis and no malignancy (Figure 3e). Five months after the biopsy, MRI showed a new 10 mm lesion contiguous to the scar zone of the biopsy (Figure 3f). The patient underwent liver transplantation, and HCC was confirmed via histology from the explanted liver (Figure 3h).

No adverse events were reported in five patients, while in four patients adverse events reported were diarrhea, softer or watery stools, and abdominal cramps (Table 3). Three patients (patients 3, 4, and 7) who had been taking fat-soluble vitamin supplements before starting odevixibat were no longer taking them at their last assessment during odevixibat treatment (Table 4). The number of vitamin supplements and/or the dose of vitamin supplements was reduced for patients 1, 5, and 6 after starting odevixibat, and patient 2 had no change in fat-soluble vitamin supplementation during treatment with odevixibat (Table 4). Patients 8 and 9 were not receiving fat-soluble vitamin supplementation before starting odevixibat; at his last assessment during odevixibat treatment, patient 8 was receiving vitamin K supplementation (Table 4).

In five of the nine patients (patients 3, 4, 5, 7, and 8), ultrasound imaging revealed no signs of disease progression during the real-world treatment period, as indicated by assessments of liver parenchyma, liver stiffness (elastography/acoustic radiation force impulse [ARFI] imaging), and/or spleen size (Table S2). Patients 3, 5, and 7, who presented with hepatomegaly and/or splenomegaly prior to treatment, were subsequently within normal ranges following odevixibat treatment; however, splenomegaly worsened despite odevixibat treatment in patients 6 and 9. In patients 1 and 2, who did not respond to odevixibat treatment, disease progression was observed on ultrasound, with evidence of focal lesions and increasing fibrosis (e.g., increasing heterogeneity of liver parenchyma in both patients and increasing elastography values in patient 2). Analysis of explanted livers from patients 1 and 2 confirmed lack of BSEP protein expression and showed bridging fibrosis (Ishak stage 5/6) in patient 1 and portal fibrosis (Ishak stage 2/6) in patient 2.

Odevixibat administration was rated as “easy” or “very easy” for all but one patient (Table 3). Some patients swallowed the odevixibat beads with water, or the beads were mixed with foods such as yogurt, muesli, or stewed fruit. Administration was rated as “difficult” in one patient, for whom odevixibat was administered via PEG tube. Generally, there was no difference in ease of administration between older and younger patients.

## 4. Discussion

Prior to the availability of IBAT inhibitors (including odevixibat) for the treatment of pruritus in patients with PFIC, pruritus therapy had been an unmet need, as many other treatments have been used off label and are frequently ineffective [9,10]. Surgical biliary diversion (SBD) is an effective yet invasive procedure that carries risks and is not universally accepted; hence, many patients with PFIC ultimately receive liver transplantation due to unrelenting pruritus [8,9,11,18]. In a previous case report, pharmacologic therapy with odevixibat achieved reductions in serum bile acids and improvements in pruritus and sleep similar to SBD in a patient with PFIC [18].

This single center cohort includes the longest reported duration of follow-up of real-world odevixibat treatment in patients with PFIC [19,20,21]. Of the nine patients in this case series, seven had positive outcomes with odevixibat, including reduction in serum bile acid levels and liver function tests, as well as improvements in pruritus and sleep disturbance. Because results from the NAPPED database suggest a relationship between decreased serum bile acid levels and improved native liver survival (NLS) in patients with PFIC1 and PFIC2 after SBD [4,6], the reductions in serum bile acids observed with odevixibat in our patient cohort are suggestive of beneficial long-term prognosis, including potential improvement in NLS; however, longer-term results are needed to confirm this finding. Additionally, analyses of individual bile acid species in odevixibat treatment responders with PFIC2 from a clinical trial show that odevixibat may improve hepatobiliary secretion in patients who respond to treatment [22]; however, further analyses would need to be performed to determine the individual bile acid profile of treatment responders in our cohort.

Previous research has shown that odevixibat is minimally absorbed and acts selectively in the terminal ileum with minimal systemic exposure [23]. Consistent with this profile, the most common adverse events observed with odevixibat in this real-world cohort, as well as in odevixibat clinical trials in healthy subjects and patients with PFIC [12,16,17,24], have been gastrointestinal events, most of which were transient or resolved with a dose reduction.

The PEDFIC 1 and 2 studies have demonstrated prolonged efficacy of odevixibat on serum bile acid level reductions, pruritus improvement, and sleep improvement in patients with PFIC [16,17]. Patient 4 did not participate in the PEDFIC trials; thus, the entire duration of odevixibat treatment for this patient was observed in this center. This patient experienced a rapid reduction in serum bile acid levels from 246 µmol/L to 10 µmol/L within approximately 1 month of initiating odevixibat that was sustained over 10.5 months of treatment. Pruritus and sleep disturbance also improved within approximately 1 month of treatment, with additional improvements over time.

PFIC can present in an episodic form characterized by intermittent cholestatic attacks; episodic cholestasis can also be caused by mutations in PFIC-associated genes (e.g., *ATP8B1* and *ABCB11*) [25]. Contrary to the rapid onset of beneficial effects of odevixibat observed in patients with persistent symptoms who responded to odevixibat treatment (patients 3–7), the two patients with PFIC2 in our cohort who experienced periodic relapses of symptoms as a part of the natural course of their disease required a longer treatment duration to achieve symptom control; in these patients (patients 8 and 9), improvements in pruritus and sleep disturbance were not reported until approximately 2 and 4 months of odevixibat treatment, respectively. Results in these two patients are consistent with the positive outcomes previously reported in patients with episodic cholestasis and *ATP8B1* mutations who were treated with odevixibat [26]. Our results suggest that approximately 3 months of treatment with odevixibat may be the minimal treatment duration needed to evaluate treatment response.

Fat-soluble vitamin deficiencies and consequent growth failure are commonly reported in patients with PFIC [1,2,9]. Deficiencies in vitamins A, D, E, and/or K often necessitate nutritional support and/or vitamin supplementation [2]; in our dataset, one patient started a new vitamin supplement during treatment with odevixibat and the other eight patients either stopped taking supplements, had reductions in their supplement regimen, or remained on pre-odevixibat supplement doses. Although median reductions in height and weight Z scores were observed in this cohort, effect sizes were small and the duration of treatment limited; indeed, improved growth has been reported with longer-term odevixibat treatment in patients with PFIC enrolled in the PEDFIC 2 study [17,24]. Additional data on odevixibat treatment in patients with PFIC over a longer treatment duration are needed to better characterize any changes in growth associated with odevixibat treatment, including both the timing and magnitude of effects.

The two patients in our cohort with PFIC2 with BSEP3 subtype mutations and total lack of BSEP expression (patients 1 and 2) did not respond to treatment with odevixibat and underwent liver transplantation. These results are consistent with previous work showing that patients with PFIC2 with BSEP3 subtype mutations generally have worse outcomes related to native liver survival (even after undergoing SBD) compared with patients with BSEP1 and BSEP2 subtype mutations [4]. In fact, effectiveness of odevixibat likely requires some residual function of BSEP protein [13,14]. Patients with BSEP3 variants should be monitored closely regarding response to treatment, evolution of liver function, and screening for HCC [4,27].

Screening for HCC remains important in patients with PFIC, despite control of symptoms, and particularly in patients with PFIC2 with BSEP3 subtype mutations, as in the case of one patient in our cohort. Patients with PFIC2 with BSEP3 subtype mutations have higher incidence of HCC compared with less severe genotypes [4], necessitating careful screening in these patients via twice-yearly AFP tests and ultrasound [2,27]. Follow-up MRI may be needed for more sensitive evaluation of questionable lesions [27].

A limitation of this cohort study is that standardized pruritus and sleep disturbance assessment tools were not used at consistent time points for each patient throughout the observation period. In addition, our pruritus and sleep results cannot be directly compared with results of clinical trials with odevixibat because different assessment tools are used in each study.

## 5. Conclusions

In our center, odevixibat was effective in patients with PFIC1 and PFIC2 (except in the cases of PFIC2 with BSEP3 subtype mutations), leading to control of symptoms and improvements in sleep, as well as improvements in cholestasis as measured by serum bile acid levels. In some patients, especially those with periodic relapses of symptoms, a minimum odevixibat treatment period of approximately 3 months may be required to observe symptom control. Patients with PFIC2 with BSEP3 subtype variants and patients without BSEP expression in biopsies require careful monitoring focused on symptom control, liver function, AFP levels, and adequate imaging to detect early development of HCC. The effectiveness and feasibility of odevixibat in our cohort demonstrate the potential for long-term benefits with odevixibat in real-world treatment of patients with PFIC.

## Figures and Tables

**Table 1:** Patient demographics, medical history, and baseline characteristics.

Patient	Sex	Age at Last Assessment, Years	Age at Genetic Diagnosis, Years	Diagnosis	Gene (DNA Mutations)	Medical History, Leading Signs and Symptoms, and Procedures	Maximum Symptom Severity Before Odevixibat ^a^		Peak ^a^ sBA, μmol/L	Peak ^a^ TB, mg/dL	Peak ^a^ ALT/ AST, U/L
							Pruritus	Sleep Disturbance			
1	M	1.8	0.1	PFIC2 (BSEP3) ^b^	*ABCB11* (c.1966_1967delTT; c.3722T>C)	Neonatal cholestasis; jaundice, failure to thrive; PEG ^c^	Severe	Mild	468	16.5	768/ 938
2	F	6.4	0.5	PFIC2 (BSEP3) ^b^	*ABCB11* (c.1966_1967delTT; c.3722T>C)	Vitamin K deficiency hemorrhage; apathetic, vomiting, failure to thrive; PEG ^c^	Severe	Severe	236	6.9	398/ 538
3	M	5.3	1.1	PFIC2	*ABCB11* (c.992C>T; c.640G>A)	Jaundice	Severe	Severe	527	16.8	60/ 135
4	M	1.3	0.3	PFIC2	*ABCB11* (c.409G>A; c.833G>A)	Neonatal cholestasis; intermittent discolored stools, jaundice	Severe	Severe	260	4.6	196/ 191
5	M	4.9	0.7	PFIC2	*ABCB11* ^d^ (c.1445A>G; c.3083C>T)	Vitamin K deficiency hemorrhage; transaminase elevation	Severe	Severe	306	2.2	191/ 144
6	M	18.8	15.1	PFIC1	*ATP8B1* ^e^ (c.1982T>C; c.2097+2T>C)	Cholestasis with low GGT; jaundice, fatigue, vomiting, not able to perform; nasobiliary tube ^f^	Severe	Severe	362	13.1	235/ 158
7	F	6.6	0.6	PFIC1	*ATP8B1* (c.2854C>T; c.2600G>A)	Jaundice	Severe	Severe	373	10	102/ 120
8	M	15.8	~1	PFIC2 ^b^	*ABCB11* (c.278A>C; c.463A>C)	Neonatal cholestasis; jaundice	Severe ^g^	Severe ^g^	433	12.5	97/ 57
9	M	22.9	9.2	PFIC2	*ABCB11* (c.278A>C; c.463A>C)	Transient neonatal cholestasis, coagulation disorder; jaundice; nasobiliary tube ^h^	Severe ^g^	Severe ^g^	467	10.8	322/ 186

^a^ Maximum severity or highest recorded value before any odevixibat treatment. ^b^ Complete loss of BSEP protein expression. ^c^ Currently in use. ^d^ Heterozygous mutation in MDR3 and homozygous and heterozygous mutations in FIC1 were also detected. ^e^ Heterozygous mutation in MDR3 was also detected. ^f^ In place from November 2018–December 2018; no subsequent symptoms until July 2020. ^g^ Patient experiences infection-triggered relapses. ^h^ In place temporarily in March 2018 and August 2022; resulted in almost complete pruritus improvement. ALT, alanine aminotransferase; AST, aspartate aminotransferase; BSEP, bile salt export pump; F, female; FIC1, familial intrahepatic cholestasis 1; GGT, gamma-glutamyl transferase; M, male; MDR3, multidrug resistance protein 3; PEG, percutaneous endoscopic gastrostomy; PFIC, progressive familial intrahepatic cholestasis; sBA, serum bile acid; TB, total bilirubin.

**Table 2:** Treatment with odevixibat.

Patient	Observation Time Before Starting Odevixibat or Entering into the PEDFIC Trials, Years	Time in PEDFIC Trials, Months	Age at Start of Real-World Odevixibat, Years	Odevixibat Treatment Duration Outside of PEDFIC Trials ^a^, Months	Starting Dose ^b^, µg/kg/d	Current Dose ^a^, µg/kg/d
1	0.9	−	1	12	50	100 ^c^
2	1.8	5 (PEDFIC 1) 22 (PEDFIC 2)	5.7	9	40	40
3	0.5	31 (PEDFIC 1 + 2)	4.1	11	130	125
4	0.3	−	0.5	11	40	120
5	1.1	30 (PEDFIC 1 + 2)	3.8	12	115	100
6	2.3	17 (PEDFIC 2)	18.2	5	40	33
7	1.8	41 (PEDFIC 2)	5.5	14	120	120
8	14.4	−	15	10	40	40
9	13.1	−	21.7	15 ^d^	40	100 ^e^

^a^ At time of last assessment outside of PEDFIC trials. ^b^ Outside of PEDFIC trials. ^c^ Dose was escalated from 50 µg/kg/d to 100 µg/kg/d to 120 µg/kg/d and then de-escalated to 50 µg/kg/d and re-escalated to the current dose. ^d^ Includes a 4-month interruption of treatment. ^e^ Patient discontinued treatment at a dose of 40 µg/kg/d due to resolution of symptoms; treatment was restarted 4 months later due to relapse of symptoms with a dose escalation to the current dose.

**Table 3:** Physician and caregiver-reported outcomes in patients with PFIC following treatment with odevixibat.

Patient	Physician-Reported Change in Signs and Symptoms After Odevixibat	Physician and/or Caregiver-Reported Adverse Events or Side Effects	Caregiver-Reported Outcomes			Caregiver-Reported Ease of Administration
			Global Satisfaction (0–4) ^a^	Current Pruritus Assessment (VAS 0–10) ^b^	Current Sleep Disturbance	
1	Consistent pruritus improvement Stools a little more fluid Liver transplantation ^c^	None	3	3–4	From time to time	Easy
2	No consistent pruritus improvement Liver transplantation ^d^	None	1	6–8	Yes	Difficult ^e^
3	Completely resolved symptoms Normal everyday life	Slightly more liquid stool 1–2×/day	4	0	No	Very easy
4	Significant reduction in pruritus	None	4	0	No	Very easy
5	Pruritus and sleep disturbance absent No evidence of GI side effects	None	4	0	No	Easy
6	All symptoms ^f^ completely gone	Diarrhea 1–2×/day	4	0	No	Very easy
7	No pruritus Good thriving Softer stool consistency	Softer stool consistency Stool 2–3×/day	4	0	No	Easy
8	No pruritus Protracted hyperbilirubinemia (3–10 mg/dL) Infection-triggered relapses	None	4	0	No	Very easy
9	Pruritus and sleep disturbance absent Diarrhea 1–2× daily Infection-triggered relapses	Transient abdominal cramps and watery stools; no complaints after introductory phase	4	0	No	Very easy

^a^ 0 = not at all satisfied, 4 = very satisfied. ^b^ 0 = no itch, 10 = worst imaginable itch. ^c^ Due to HCC manifestation. ^d^ Due to severe failure to thrive and refractory pruritus. ^e^ Patient is fed completely via PEG; beads were reported as leading to a blockage of PEG tube. ^f^ Fatigue, vomiting, itching, and sleep disturbance. GI, gastrointestinal; HCC, hepatocellular carcinoma; PEG, percutaneous endoscopic gastrostomy; PFIC, progressive familial intrahepatic cholestasis; VAS, visual analog scale.

**Table 4:** Fat-soluble vitamin supplement dose changes with odevixibat.

Patient	Vitamin Supplements Before Starting Any Odevixibat Treatment		Vitamin Supplements During Odevixibat Treatment	
	Vitamin	Dose	Vitamin	Dose
1	DEKAs Vitamin Essential ^a^	1 × 1 mL	DEKAs Vitamin Essential ^a^	1 × 0.5 mL
2	Vitamin D	1000 IE	Vitamin D	1000 IE
	Vitamin E	111 IE	Vitamin E	111 IE
	Vitamin K	3 mg 3×/week	Vitamin K	3 mg 3×/week
3	Vitamin A	3 × 4000 IE	None	NA
	Vitamin D	500 IE		
	Vitamin E	3 × 111 IE		
	Vitamin K	2 mg 1×/week		
4	Vitamin A	7500 IE	None	NA
	Vitamin D	3000 IE		
	Vitamin E	105 IE		
	Vitamin K	3 mg		
5	Vitamin A	1 × 20,000 IE	Vitamin A Vitamin D Vitamin K	1 × 20,000 IE 1 × 2000 IE 2 mg 3×/week
	Vitamin D	3000 IE daily		
	Vitamin E	1 × 225 IE		
	Vitamin K	2 mg 3×/week		
6	Vitamin D	2000 IE	Vitamin D	2000 IE
	Vitamin K	5 mg		
7	Vitamin D	2000 IE	None	NA
8	None	NA	Vitamin K	1 × 10 mg
9	None	NA	None	NA

^a^ Ingredients per mL: vitamin D, 1818 IU; vitamin K, 1.8 mg; vitamin A (as palmitate and beta-carotene), 2273 IU; vitamin E (as tocofersolan), 75 IU. DEKAs is a registered trademark of Alveolus Biomedical BV. NA, not applicable.

## Data Availability

The datasets generated during the current study are not publicly available due to patient privacy concerns, but anonymized data are available from the corresponding author on reasonable request.

## Supplementary Materials

The following supporting information can be downloaded at: https://www.mdpi.com/article/10.3390/jcm13247508/s1.
