# Supplementary material for: Practical Considerations for Odevixibat Treatment in Patients with Progressive Familial Intrahepatic Cholestasis: A Single-Center Case Series
Source: J Clin Med. 2024 Dec 10;13(24):7508. doi: 10.3390/jcm13247508 (PMC11676709; doi:10.3390/jcm13247508)
Supplement: Supplementary file 1 [file jcm-13-07508-s001.zip › jcm-3292282-supplementary.pdf]

Supplementary Files

Plain Language Summary

Patients with a disease called progressive familial intrahepatic cholestasis (PFIC) develop liver disease and can have uncontrollable itching that can disturb their sleep and daytime activities. In PFIC, the movement of bile acids out of the liver is disrupted (bile acids are small soap-like molecules that are made in the liver and help digest fats in the intestines). As well as increased bile acids being kept in the liver, this can cause increased bile acids in the blood (called serum bile acids).

Medicines for PFIC are limited. Odevixibat is a medication that improved scratching and decreased serum bile acids in patients with PFIC during clinical trials. This study describes 9 patients with PFIC who were treated with odevixibat in Tübingen, Germany. For 5 patients (3 patients with PFIC type 2 and 2 patients with PFIC type 1), odevixibat treatment improved scratching, sleep, and liver function tests and decreased serum bile acids. Improvements with odevixibat were also seen in 2 patients with PFIC type 2 who had periodically occurring PFIC symptoms; however, it took these patients until 2 to 4 months to see results. Two other patients who had a more severe form of PFIC type 2 (called BSEP deficiency type 3) did not show response to odevixibat and had liver transplantations. Four of the 9 patients had side effects that included stomach cramps and/or diarrhea; these either did not last long or went away when the medication dose was decreased.

Results from this study suggest that real-world odevixibat treatment could provide long-lasting benefits for patients with PFIC and that patients with severe disease need careful monitoring.

Figure S1. Standardized Case Report Form

Patients With Progressive Familial Intrahepatic Cholestasis (PFIC)

Case Report Form for publication purpose

Patient ID:
Initials:

Hospital

Healthcare Professional:	Date (MM/DD/YYYY):

Patient Demographics

Sex: ☐ Male ☐ Female	Date of birth:<br>Current age:<br>Current Weight:<br>Current Height:

Medical and Surgical History

Diagnosis:

Date of symptom onset:

Symptoms:

Date of diagnosis:

Genetic information:

Gene	Mutation/Variant	Homozygous/ heterozygous	Mutation of protein	origin	Other mutations

All rights reserved. Albireo Pharma, Inc.

Histological information:

Liver biopsy available: ☐ Yes ☐ No

Please describe any previous disease-related surgeries or procedures, with dates:

For any interventions noted above, please describe the intervention's efficacy and whether it was permanent or removed:

Observation period:

Trial participation (study code)

In this section, please consider the clinical situation immediately <u>before</u> initiation of odevixibat therapy. Please supply the clinical details listed below.

Medications

Please list medication for pruritus, vitamins and any medications, including dose and approximate duration of use ...)

Laboratory Parameters before the Start of odevixibat treatment. Please add all data you have, as early in the disease course as possible

Date 1:

Date 2:

Date 3:

Date 4:

Date 5:

Date 6:

Date 7:

All rights reserved.

	Date 1 of FU before treatment	Date 2 of FU before treatment	Date 3 of FU before treatment	Date 4 of FU before treatment	Date 5 of FU before treatment	Date 6 of FU before treatment	Date 7 of FU before treatment	Date 8 of FU before treatment
Alanine aminotransferase, U/L								
Aspartate aminotransferase U/L								
Total bilirubin, mg/dl								
Conjugated bilirubin mg/dl								
Serum bile acids (total fasting), µmol/L								

All rights reserved. Albireo Pharma, Inc.

	Date 1 of FU before treatment	Date 2 of FU before treatment	Date 3 of FU before treatment	Date 4 of FU before treatment	Date 5 of FU before treatment	Date 6 of FU before treatment	Date 7 of FU before treatment	Date 8 of FU before treatment
Prothrom-bin Index , %								
Albumin, g/dl								
Factor V								
Hemoglobin g/dl								
Alpha-foetopro-tein, µg/l								
Platelets thousand/µl								
White cells , 1/µl								
Vitamin<br>-A, µmol/l<br>-D (25-OH-Vit D), nmol/l<br>-E, , µmol/l								
Cholesterol levels, mg/dl								

All rights reserved.

Triglycerides, mg/dl								
AP, U/l								
Gamma-GT, U/l								
Cholinesterase, kU/l								

Symptoms of Interest

Please describe any patient-reported pruritus or sleep disturbance prior to starting Odevixibat treatment. Please add all data you have, as early in the disease course as possible

	Pruritus<br><br>Please enter data according to the assessment method you have used. If none, please rate as severe/moderate/mild/absent	Sleep disturbance<br><br>Please enter data according to the assessment method you have used. If none, please rate as severe/moderate/mild/absent
Date 1 of FU before treatment		

Ultrasound performed before treatment (dates and outcome)

Elastometry performed before treatment (dates and outcome)

Comments

Please record any other additional data of interest:

Odevixibat Dosing

Reason(s) for starting odevixibat:

Intractable pruritus : ☐ Yes ☐ No

If no, please add comments

Treatment start date:

Starting dose:	Frequency:

Have there been any dose changes? If yes, please describe:

Have there been a re-escalation in odevixibat dose ? ☐ Yes ☐ No

If yes, please indicate date :

Dose of re-escalation:

Clinical Details <u>During</u> Odevixibat Therapy

Concomitant Medications

Please list any medications, including dose and approximate duration of use:

All rights reserved. Albireo Pharma, Inc.

Laboratory Parameters – Values in the First Months after staring Odevixibat Therapy. **Please add all data you have,**

Date 1:

Date 2:

Date 3:

Date 4:

Date 5:

Date 6:

Date 7:

Date 8:

	Date 1 of FU after treatment	Date 2 of FU after treatment	Date 3 of FU after treatment	Date 4 of FU after treatment	Date 5 of FU after treatment	Date 6 of FU after treatment	Date 7 of FU after treatment	Date 8 of FU after treatment
Alanine aminotransferase, U/L								
Aspartate aminotransferase U/L								
Total bilirubin, mg/dl								
Conjugated bilirubin mg/dl								
Serum bile acids (total fasting), μmol/L								

All rights reserved.

	Date 1 of FU after treatment	Date 2 of FU after treatment	Date 3 of FU after treatment	Date 4 of FU after treatment	Date 5 of FU after treatment	Date 6 of FU after treatment	Date 7 of FU after treatment	Date 8 of FU after treatment
Prothrombin Index, %								
Albumin, mg/dl								
Factor V								
Hemoglobin, g/dl								
Alpha-foetoprotein, µg/l								
Platelets , tsd/µl								
White cells , 1/µl								
Vitamin<br>-A, µmol/l<br>-D (25-OH-Vit D), nmol/l<br>-E, µmol/l								

All rights reserved.

Cholesterol levels, mg/dl								
Triglycerides , mg/dl								
AP, U/l								
Gamma-GT, U/l								
Cholinesterase, kU/l								

Clinical Parameters – Values in the First Months after treatment

	Odevixibat dose	**Pruritus** Please enter data according to the assessment method you have used. If none, please rate as severe/moderate/mild/absent	**Sleep disturbance** Please enter data according to the assessment method you have used. If none, please rate as severe/moderate/mild/absent
date 1 since staring odevixibat			

Ultrasound performed after treatment (dates and outcome)

Elastometry performed after treatment (dates and outcome)

All rights reserved. Albireo Pharma, Inc.

Symptoms of Interest

Please describe any impacts the patient had noted in his/her daily life:

Please record any other additional data of interest:

Interview with parents (date)

1. How easy was administration of Odevixibat?

   →

2. Were/Are there any problems with Odevixibat? Were/Are there any adverse side effects?

   →

3. How is the global satisfaction with Odevixibat (0-4, 0=not at all satisfied, 4= very satisfied)? Current Pruritus assessment (VAS 0-10) and assessment of sleep disturbance (Yes/No)?

   →

Reported Adverse Events

Reporting of Suspected Adverse Reactions

Reporting suspected adverse reactions after authorization of the medicinal product is important. It allows continued monitoring of the benefit/risk balance of the medicinal product. Healthcare professionals are asked to report any suspected adverse reactions via their respective national reporting system listed in Appendix V, which is included as an icon here.

All rights reserved. Albireo Pharma, Inc.

Appendix V

Please return the completed form to ....

All rights reserved. Albireo Pharma, Inc.

Table S1. Weight and Height Z Scores Before and After Real-World Odevixibat Treatment

Patient	Weight Z Score Before Real-World Odevixibat	Weight Z Score After Odevixibat[a]	Change in Weight Z Score	Height Z Score Before Real-World Odevixibat	Height Z Score After Odevixibat[a]	Change in Height Z Score
1	–1.71	–0.72[b]	0.99	–1.86	–2.82[b]	–0.96
2	–2.77	–2.95[b]	–0.18	–2.95	–3.02[b]	–0.07
3	0.43	0.37	–0.06	0.62	0.23	–0.39
4	0.95	–0.06	–1.01	0.20	–0.05	–0.25
5	–0.08	–0.05	0.03	0.02	–0.32	–0.34
7	–0.44	–1.10	–0.66	–2.08	–1.79	0.29
8	–0.30	–0.12	0.18	0.52	0.29	–0.23
**Median (Range)**			**–0.06** (–1.01, 0.99)			**–0.25** (–0.96, 0.29)
**Median (Range) in Responders[c]**			**–0.06** (–1.01, 0.18)			**–0.25** (–0.39, 0.29)

No Z scores appear for patients 6 and 9 because they were >18 years old during real-world odevixibat treatment. [a]At last assessment on odevixibat treatment. [b]Before liver transplantation. [c]Includes patients who were responders and who had available weight and height Z scores (patients 3–5, 7, and 8).

Table S2. Ultrasound Findings Before and After Real-World Odevixibat Treatment

Patient	Before Starting Any Odevixibat Treatment	After Odevixibat Treatment
1	Hepatomegaly	Focal lesion in segment VII
2	Unremarkable	Cholecystolithiasis<br>Increased liver stiffness/shear wave velocity<br>Heterogeneous parenchyma
3	Hepatomegaly	Liver size normal
4	Unremarkable	Elastography normal
5	Splenomegaly (7.4 cm)<br>Liver unremarkable	Spleen upper normal range (90th percentile)<br>Elastography normal
6	Splenomegaly (12.9 cm, slightly >95th percentile)	Splenomegaly (14 cm, >95th percentile)<br>Elastography unremarkable
7	Hepatomegaly	No abnormalities
8	Unremarkable	Spleen unremarkable<br>Elastography slightly increased
9	Liver unremarkable<br>Spleen, 12.8 cm	Hepatosplenomegaly (17 cm)<br>Spleen enlarged (14.7 cm)
